# Phenotypic Plasticity in the Structure of Fine Adventitious *Metasequoia glyptostroboides* Roots Allows Adaptation to Aquatic and Terrestrial Environments

**DOI:** 10.3390/plants8110501

**Published:** 2019-11-14

**Authors:** Chaodong Yang, Xia Zhang, Ting Wang, Shuangshuang Hu, Cunyu Zhou, Jian Zhang, Qingfeng Wang

**Affiliations:** 1 The College of Horticulture and Gardening, Yangtze University, Jingzhou 434025, Hubei, China; yangcd22@163.com (C.Y.); zhangxia20192018@163.com (X.Z.); wt201875107@163.com (T.W.); 201872480@yangtzeu.edu.cn (S.H.); zhoucy@yangtzeu.edu.cn (C.Z.); zhangjian840000@163.com (J.Z.); 2 Key Laboratory of Aquatic Botany and Watershed Ecology, Wuhan Botanical Garden, The Chinese Academy of Sciences, Wuhan 430074, Hubei, China

**Keywords:** air spaces, dilated parenchyma, endodermis, exodermis, cork, lignified Φ thickenings

## Abstract

*Metasequoia glyptostroboides* (Cupressaceae) is a rare deciduous conifer which grows successfully in both aquatic and terrestrial environments. This tree has a narrow natural distribution in central China but is cultivated worldwide. Using histochemical staining and microscopy (both brightfield and epifluorescent), we investigated whether the phenotypic anatomical and histochemical plasticity in the fine adventitious roots of *M. glyptostroboides* has promoted the adaptation of this plant to aquatic and terrestrial environments. The fine root development and cortex sloughing of *M. glyptostroboides* occurs later in aquatic habitats than in terrestrial habitats. Anatomical and histochemical analyses have revealed that the apoplastic barriers in the primary growth of the fine roots consist of the endodermis and exodermis with Casparian bands, suberin lamellae, and secondarily lignified cell walls. There were also lignified phi (Φ) thickenings in the cortex. In both aquatic and terrestrial roots, secondary growth was observed in the vascular cambium, which produced secondary xylem and phloem, as well as in the phellogen, which produced cork. As compared to terrestrial adventitious roots, aquatic adventitious roots had multiple lignified Φ thickenings throughout the cortex, larger air spaces, dilated parenchyma, and dense suberin and lignin depositions in the exodermis. Our results thus indicate that phenotypic plasticity in the anatomical features of the fine adventitious roots, including apoplastic barriers, air spaces, and lignified Φ thickenings, might support the adaptation of *M. glyptostroboides* to both aquatic and terrestrial environments.

## 1. Introduction

Although *Metasequoia glyptostroboides* (Cupressaceae) is a rare deciduous conifer, with a natural distribution limited to small areas of central China, including western Hubei, northern Hunan and eastern Chongqing [1,2], this species is now commonly cultivated worldwide [3]. *M. glyptostroboides* was compared to the fossil *M. milleri* by Basinger [4]. The morphology of *M. glyptostroboides* and the structures of its fine adventitious roots, cuticle, and intertracheary pit membranes have been well studied [4,5,6,7,8,9]. The root structures in other gymnosperms, such as *Thuja occidentalis*, *Libocedrus decurrens*, *Cunninghamia lanceolata*, and *Ginkgo biloba* have been extensively investigated [10,11,12,13,14]. Lignified phi (Φ) thickenings have been recorded in the inner cortex adjacent to the endodermis in *M. milleri* [4] and are considered similar to the endodermis in the roots of *C. lanceolata*, *M. glyptostroboides*, and other gymnosperm species [9,14,15]. More broadly, lignified Φ thickenings have been reported in the root cortices of several species, including the gymnospermous *Ginkgo biloba* [13] and *C. lanceolata* [14], and the angiospermous *Pyrus malus* [16], *Brassica napus*, *B. oleracea* [17,18], *Myrica rubra* [19], and *Cardamine hupingshanensis* [20].

*M. glyptostroboides* grows successfully in both aquatic and terrestrial environments [9,21]. Aquatic and wetland plants are typically subjected to anoxia following flooding or submersion [22,23,24,25]. In addition to the endodermis, aquatic or wetland plants have evolved various key structural features, including aerenchyma and apoplastic barriers, such as an exodermis (with Casparian bands, suberin lamellae, and lignified secondary walls) and suberized phellem. These structures block the diffusion of air and ions [25,26,27,28,29,30,31,32,33,34,35].

It is well known that cultivation conditions can induce the growth of aerenchyma and apoplastic barriers in the roots of several angiosperm species, including rice (*Oryza sativa*) [25,26,36], maize (*Zea mays*) [37,38], and wheat (*Triticum aestivum*) [39]. Furthermore, dilation and additional cell divisions have been observed in the root cortex, secondary phloem, (e.g. the endodermis and exodermis), and aged root parenchyma of the angiosperms alligator weed (*Alternanthera philoxeroides*), *Artemisia selengensis*, *Gentiana asclepiadea*, and *Tilia americana* [34,35,40,41]. However, it is unclear whether habitat triggers aerenchymal and apoplastic barrier growth in the roots of gymnosperms.

In this study, we aimed to determine if the structural and histochemical features of the fine roots of *M. glyptostroboides* were consistent with adaptations to aquatic and terrestrial environments. Evidence of such adaptive characteristics could help to explain the ability of *M. glyptostroboides* to grow in both aquatic and terrestrial habitats. The present work may improve our understanding of the mechanisms supporting the survival of this rare plant in limited areas, and may inform future studies of taxonomy, evolution, and phylogeny in related plants.

## 2. Results

### 2.1. General Structure

**Figure 1:**
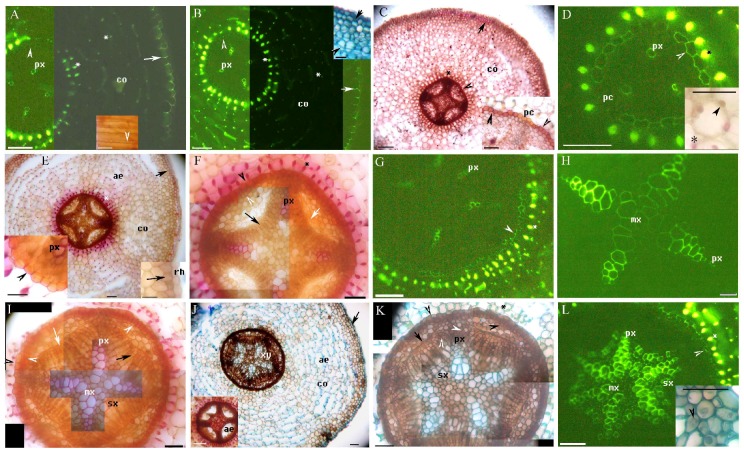
A–L. Photomicrographs of the adventitious roots of *Metasequoia glyptostroboides*, grown in aquatic environments. Roots are 150–250 mm long. Scale bars = 50 μm, except where noted. (**A**) Endodermal Casparian bands (arrowhead), protoxylem, cortex, lignified Φ thickenings (*), and exodermal Casparian bands (arrow); stained with BAB. Inset shows exodermal Casparian bands (arrowhead); visualized using sulfuric acid digestion. Scale bars = 25 μm. (**B**) Endodermal Casparian bands (arrowhead), protoxylem, cortex, lignified Φ thickenings (*), and exodermal Casparian bands (arrow); stained with berberine hemisulfate-aniline blue (BAB). Inset shows dilated cortex (arrowhead) and exodermis (arrow); stained with toluidine blue O (TBO). Scale bars = 25 μm. (**C**) Endodermis (arrowhead), cortex, Φ thickenings (*), and exodermis (arrow). Inset shows suberized endodermis (arrowhead) and passage cells; stained with Sudan red 7B (SR7B). Scale bars = 25 μm. (**D**) Protoxylem, suberized endodermis (arrowhead), passage cells, and lignified Φ thickenings (*); stained with BAB. Inset shows Φ thickenings (*) and mycorrhizal spores (arrowhead) in the cortex; stained with phloroglucinol-HCl (Pg). Scale bars = 25 μm. (**E**) Cortex, lignified Φ thickenings (*), aerenchyma, and exodermis (arrow). Left inset shows lignified endodermis (arrowhead) and protoxylem. Right inset shows lignified exodermis (arrow) and rhizodermis; stained with Pg. Scale bars = 25 μm. (**F**) The stele of Figure 1E, showing protoxylem, endodermis (black arrowhead), divided pericycle (white arrowhead), lysigenous primary phloem (white arrow), vascular cambia (black arrow), and Φ cells in cortex (*); stained with Pg. (**G**) The stele of Figure 1E, showing protoxylem, new cork (arrowhead) just one cell layer under old endodermis, and Φ cells in cortex (*); stained with BAB. (**H**) Protoxylem and metaxylem; stained with BAB. (**I**) The stele of Figure 1H, showing early secondary xylem, protoxylem, metaxylem, endodermis (black arrowhead), divided pericycle (white arrowhead), lysigenous primary phloem (white arrow), and vascular cambia (black arrow); stained with Pg. (**J**) Xylem, cortex, aerenchyma, and exodermis (arrow); stained with TBO. Inset shows aerenchyma in primary phloem; stained with Pg. Scale bars = 25 μm. (**K**) The stele of Figure 1J, showing protoxylem, secondary xylem, endodermis (left black arrowhead), divided pericycle and cork (right black arrowhead), vascular cambia (white arrowhead), lysigenous primary phloem (black arrow), and lignified Φ thickenings (*); stained with TBO. (**L**) The stele of Figure 1J, showing early secondary xylem, protoxylem, metaxylem, new and old cork from pericycle (arrowhead), and Φ cells in cortex (*); stained with BAB. Inset shows mycorrhiza (arrowhead) in the stele; stained with TBO. Scale bars = 25 μm. Abbreviations used in the figures are as follows: Aerenchyma, ae; Berberine sulfate-aniline blue, BAB; cork, cr; cortex, co; rhizodermis, rh; secondary phloem fibers, f; HCl-phloroglucinol, Pg; metaxylem, mx; passage cells, pc; protoxylem, px; Sudan red 7B, SR7B; secondary xylem, sx; toluidine blue O, TBO; xylem, xy.

**Figure 2:**
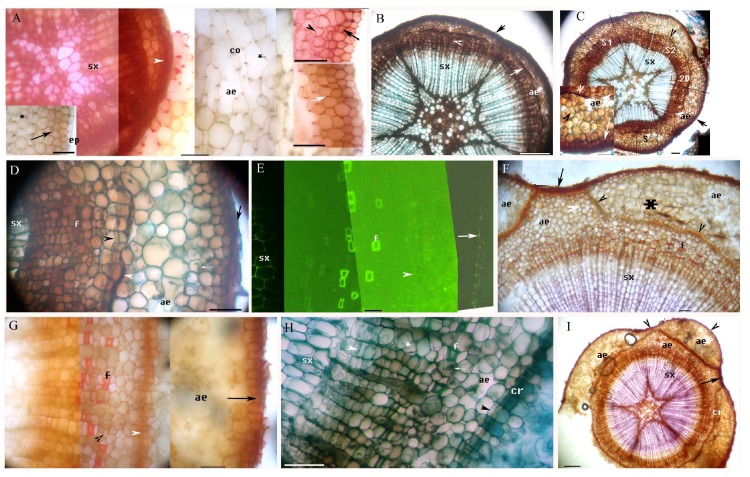
A–I. Photomicrographs of the adventitious roots of *Metasequoia glyptostroboides* grown in aquatic environments, showing the secondary growth; roots are 150–250 mm long. Scale bars = 50 μm, except where noted. (**A**) Secondary xylem, divided pericycle and cork (white arrowhead), cortex, lignified Φ thickenings (*), and aerenchyma; stained with Pg. Left inset shows exodermis (black arrow); unstaining. Right upper inset shows the suberized exodermis (black arrow) and dilated cortex (black arrowhead); stained with SR7B. Scale bars = 25 μm. (**B**) Secondary xylem, secondary phloem (white arrowhead), lysigenous primary phloem (white arrow), aerenchyma, remnant endodermis, and pericycle cork (black arrow); stained with TBO. (**C**) Secondary xylem, lysigenous primary phloem (black arrowhead), and cortical aerenchyma under remnant endodermis and pericycle cork (black arrow). Marked areas are shown magnified in other images: Area S is shown in the inset; area S1 is shown in Supplementary Figure S1; area S2 is shown in Supplementary Figure S2; area 2D is shown in Figure 2D. Inset shows the redivided cortical parenchyma (black arrowhead) under pericycle cork (white arrow), and the lysigenous primary phloem (white arrowhead); stained with TBO. (**D**) Magnification of area 2D in Figure 2C, showing secondary xylem, phellogen (black arrowhead), crushed primary phloem (white arrowhead), cortical aerenchyma, pericycle cork (black arrow), mycorrhiza (white arrow), and phloem fibers; stained with TBO. (**E**) Secondary xylem, phloem fibers, phellogen (arrowhead), and pericycle cork (arrow); stained with BAB. (**F**) Secondary xylem; aerenchyma in the secondary phloem (middle ae) under pericycle cork (left and right ae); phellogen (arrowhead) expanded to produce wing of secondary tissue and connected to the pericycle cork (arrow); dilated parenchyma (white arrowhead); parenchyma under pericycle cork (*); and phloem fibers; stained with Pg. (**G**) Magnification of subsection of Figure 2F, showing phloem fibers, phellogen (white arrowhead), dilated parenchyma in the secondary phloem (black arrowhead), aerenchyma under pericycle cork (arrow); stained with Pg. (**H**) Secondary xylem, vascular cambium (white arrowhead), phloem fibers, dilated parenchyma in the secondary phloem (*), phloem aerenchyma, mycorrhiza (white arrow), phellogen (black arrowhead), and early cork, stained with TBO. (**I**) Secondary xylem, aerenchyma in the secondary phloem (middle ae) and in parenchyma under pericycle cork (arrowhead), cork, and bark (whole arrow); stained with Pg. Scale bars = 500 μm.

**Figure 3:**
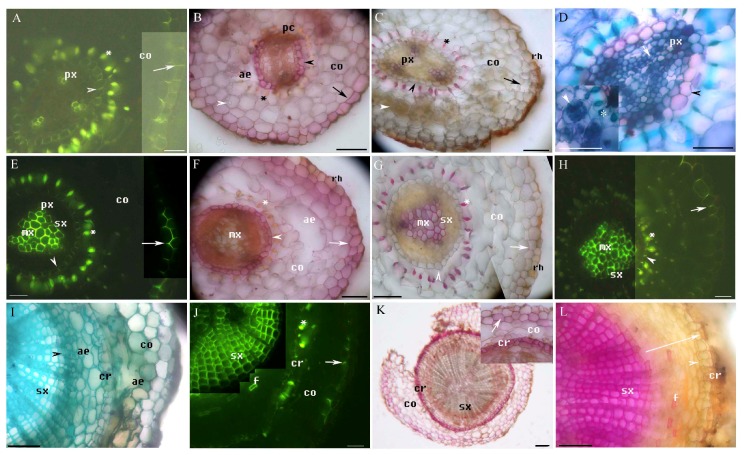
A–L. Photomicrographs of the adventitious roots of *Metasequoia glyptostroboides* grown in terrestrial environments; roots are 35–55 mm long. Scale bars = 50 μm. (**A**) Endodermal Casparian bands (arrowhead), protoxylem, cortex, lignified Φ thickenings (*), and exodermal Casparian bands (arrow); stained with BAB. (**B**) Endodermis (black arrowhead), passage cells, cortex, mycorrhizal spores (white arrowhead), aerenchyma, Φ thickenings (*), and exodermis (arrow); stained with SR7B. (**C**) Endodermis (black arrowhead), protoxylem, cortex, lignified Φ thickenings (*), mycorrhiza (white arrowhead), exodermis (arrow), and rhizodermis; stained with Pg. (**D**) Endodermis (black arrowhead), protoxylem, and cambia (arrow); stained with TBO. Inset shows Φ thickenings (*) and mycorrhiza (arrowhead) in the cortex; stained with TBO. Scale bars = 25 μm. (**E**) Secondary xylem, protoxylem, metaxylem, endodermis (arrowhead), cortex, lignified Φ thickenings (*), and exodermis (arrow); stained with BAB. (**F**) Metaxylem, endodermis (arrowhead), cortex, lignified Φ thickenings (*), aerenchyma, exodermis (arrow), and rhizodermis, stained with SR7B. (**G**) Secondary xylem, metaxylem, endodermis (arrowhead), cortex, lignified Φ thickenings (*), exodermis (arrow), and rhizodermis; stained with Pg. (**H**) Secondary xylem, metaxylem, endodermis (arrowhead), cortex, lignified Φ thickenings (*), and exodermis (arrow); stained with BAB. (**I**) Secondary xylem; vascular cambium (arrowhead); aerenchyma in the secondary phloem and cortex; phellogen and first cork; and cortex; stained with TBO. (**J**) Secondary xylem, phloem fibers, cork, lignified Φ thickenings (*), cortex, and exodermis (arrow); stained with BAB. (**K**) Secondary xylem, cork, cortex, and exodermis (arrow). Inset shows cork, remnant cortex, and exodermis (arrow); stained with SR7B. (**L**) Secondary xylem, phloem fibers, phelloderm (arrowhead), cork, and bark (whole arrow); stained with Pg.

We identified primary and secondary structures and histochemically characterized cell wall components in the adventitious roots of *M. glyptostroboides* (Table 1), including suberin (identified using Sudan red 7B staining), ligin (identified using phloroglucinol-HCl staining), Casparian bands and lignin in the cell walls (identified using berberine hemisulfate-aniline blue staining), and other structures (identified using toluidine blue O staining) [14,20,31,32,33,34,35]. The primary structure of adventitious roots included the stele, the cortex with the endodermis, the exodermis, the lignified Φ thickenings and aerenchyma, and the rhizodermis (Figure 1A–J, Figure 2A, Figure 3A–H). In the fine adventitious roots of *M. glyptostroboides* from terrestrial habitats, the cortex was observed to be closer to the root tip than in the roots from aquatic habitats (Table 1). The secondary structure of the adventitious roots contained secondary xylem, secondary phloem, aerenchyma and cork (Figure 1F–L, Figure 2A–I, Figure 3H–L). In the secondary growth stage, the suberin and lignin of the exodermis were densely located in the roots from the aquatic habitats, as compared to the roots from the terrestrial habitats (Figure 1C–F, Figure 2A, Figure 3A–C,E–G; Table 1). The cortices of the roots from the aquatic habitats had multiple lignified Φ thickenings and more spacious aerenchymatous lacunae than the cortices of roots from terrestrial habitats did (Figure 1A–C,E,J, Figure 2A, Figure 3B,C,F,G; Table 1).

### 2.2. The Structure of Adventitious Roots Grown in Aquatic Environments

The stele of the fine adventitious roots of *M. glyptostroboides* varied from the triarch to the hexarch poles of the protoxylem in aquatic environments (Figure 1A–L). At 5 mm from the root tips, the endodermis had faint Casparian bands, while the exodermis had prominent Casparian bands (Figure 1A and inset), and lignified Φ thickenings in the inner cortical cell walls (Figure 1A). In older roots, lignified Φ thickenings were also present in the outer cortex cell walls at 15–100 mm from the root apex (Figure 1J and Figure 2A). At 10 mm from the root apex, the endodermis had more prominent Casparian bands (Figure 1B), with suberin lamellae around a few passage cells (Figure 1C,D), while the exodermis had suberin lamellae and lignified secondary walls (Figure 1B,C,E and Figure 1B inset). The tracheary elements gradually matured centripetally (Figure 1A,B,D,F) until the metaxylem matured near the center (Figure 1H,I,L). The cortex had irregular schizolysigenous aerenchyma (Figure 1B inset and Figure 1C,E,J), and air spaces were observed even in the primary phloem (Figure 1J inset). The primary phloem was found between the poles of the protoxylem (Figure 1C,E). The endodermis expanded in order to add cells circumferentially (Figure 1E,G,I,J,K and Figure 1E inset). Additional Φ cells were observed in the expanded inner cortex (Figure 1G,I,K,L and Figure 2A) and other cells in the cortex dilated to expand the cortex (Figure 1B inset and Figure 1C and Figure 2A).

The pericycle over the protoxylem poles, as well as the cells between the primary xylem and the primary phloem, formed the vascular cambium, and the secondary xylem appeared at 10–15 mm from the root apex (Figure 1F,I–L). At 100 mm from the root apex, the secondary structures were observed in the stele. The cortex and its exodermis were also observed here (Figure 1I–L). The endodermis expanded in order to circumferentially increase the cells (Figure 1I–L) and developed C-shaped walls (Figure 1E,F,I,K). The exodermis had 2–3 layers of densely suberized and lignified walls (Figure 1C,E,J and Figure 2A). Lignified Φ cells were evident in the inner cortex (Figure 1I–L) and in the outer cortex (Figure 2A). There were lysigenous air spaces in the primary phloem (Figure 1J and Figure 2B,D). The cortical air space was more expanded and the exodermis was still intact (Figure 2A). The secondary xylem had radially arranged tracheary cells (Figure 1I–L and Figure 2A,B), and an additional lysigenous parenchyma was observed under the pericycle cork, forming the aerenchyma as the cortex broke up (Figure 2B–D). The aerenchymatous cortex and exodermis were not observed beyond 100–110 mm from the root apex.

A redivided pericycle also formed the phellogen, beginning the production of the cork at the outer face of the meristem (Figure 1F,G,I,K,L and Figure 2A–C). The phellogen appeared under the lysigenous primary phloem and close to the pericycle cork as flares of expanded secondary phloem (Figure 2C–E, Figure S1, Figure S2). Dilated parenchyma cells were observed under the pericycle cork (Figure 2C,F) and in the secondary phloem (Figure 2G–I). Fibers were present in the secondary phloem (Figure 2D–H). Finally, the secondary xylem was prominent in the center of the stele. The parenchyma and the aerenchyma in the secondary phloem expanded in a flare shape (Figure 2C,F,I). Mycorrhizal spores and mycorrhizae were present in the cortex (Figure 1D), stele (Figure 1L), and parenchyma of the phloem (Figure 2D,H).

### 2.3. The Structure of Adventitious Roots Grown in Terrestrial Environments

The stele of *M. glyptostroboides* grown in terrestrial environments (Figure 3) had almost exclusively diarch (Figure 3B–D) and triarch protoxylem groups (Figure 3A,C,E–G). The endodermis and exodermis had Casparian bands (Figure 3A), suberin lamellae (Figure 3B), and lignin (Figure 3C). Lignified Φ thickenings (Figure 3A) occurred primarily in the inner cortex, and small amounts of aerenchyma occurred in the cortex at 5 mm from the root tip (Figure 3B,C). At 10 mm from the root tip, the vascular cambium appeared (Figure 3D).

At 10–15 mm from the root tip, the stele had a central metaxylem (Figure 3E), followed by the secondary xylem (Figure 3E,G). Suberin and lignin were prominent in the endodermis and exodermis (Figure 3E–G). Lignified Φ thickenings and large, irregular air spaces were observed in the cortex (Figure 3F,G). At 20 mm from the root tip, the stele had progressively more secondary xylem (Figure 3H–J). Lignified Φ thickenings still characterized the inner cortex, and the exodermis characterized the outer cortex (Figure 3H,J). In addition, the phellogen produced the first cork (Figure 3I,J). At 25 mm from the root tip, the secondary xylem dominated the root, where the cortex ruptured and sloughed off (Figure 3K,L). A vascular cambium with a secondary xylem and a fibrous phloem was conspicuous (Figure 3J,L), as was a phellogen with suberized cork (Figure 3L). Some small air spaces were observed in the secondary phloem. The bark consisted of secondary phloem, phellogen, phelloderm, and cork (Figure 3L). Mycorrhizal spores and mycorrhizae were present in the cortex (Figure 3B–D).

## 3. Discussion

Although *M. glyptostroboides* from terrestrial and aquatic habitats exhibits similar anatomical and histochemical features, the cortex is sloughed off later in *M. glyptostroboides* roots from aquatic habitats. *M. glyptostroboides* roots from terrestrial and aquatic habitats have primary structures that include an endodermis, an exodermis, and lignified Φ thickenings in the cortex. In aquatic and terrestrial *M. glyptostroboides*, similar to very many other plants, roots develop a vascular cambium with secondary xylem and phloem, as well as a cork cambium, a phellogen with cork [13,27,42,43]. These cambia arise from the pericycle and the parenchyma between the primary phloem and xylem, as was observed in the fossil *M. milleri* [4]. There was no evidence of local flaring in the secondary phloem and phellogen in the terrestrial roots [4], in contrast to the aquatic roots. In the aquatic roots, this flare might serve to push phellogen and young cork through the parenchyma to the pericycle cork.

We easily identified the endodermis in both aquatic and terrestrial *M. glyptostroboides* roots. The endodermis was also identified by Basinger [4], although the modified cell walls in *M. glyptostroboides* and in the fossil *M. milleri* were not evident. In roots from aquatic habitats, the cell walls of the exodermis had dense suberin and lignin deposits, and the cortex was sloughed off later. These features were not noted by Basinger [4]. The endodermis and the exodermis deposited the Casparian bands, suberin lamellae, and lignin nearer to the tips than the adventitious roots of rice and *Zea nicaraguens* grown in stagnant water, as compared to aquatic roots of *M. glyptostroboides* [25,26,36,38]. Lignin on the rhizodermis also develops early in *Z. nicaraguensis*. In alligator weed, the cortex and the hypodermis of the aquatic roots had denser lignin depositions than the terrestrial roots did, and, unlike *M. glyptostroboides*, alligator weed had cortical lignified Φ thickenings and an exodermis [35]. The roots of wetland or aquatic eudicots, such as *Ranunculus trichophyllus*, *Hydrocotyle sibthorpioides*, *Artemisia lavandulaefolia*, and *A. selengensis*, possess an endodermis, a uniseriate exodermis, and a cortex that lacks lignified Φ thickenings [34,35,44,45]. In contrast, the roots of wetland grasses, such as *Phragmites* and *Oryza,* possess an endodermis and a multiseriate exodermis [25,26,29,30,31,32,33,36].

The cortical lignified Φ thickenings of *M. glyptostroboides* were similar to those of the gymnosperms *G. biloba* and *C. lanceolata*, but are absent in the Cycadaceae, Gnetaceae, Pinaceae, and Podocarpaceae families [9,15,17,18]. Basinger [4] identified only one layer of Φ cells in *M. milleri*. He did not illustrate Φ cells in *M. glyptostroboides. Myrica rubra*, *Pyrus malus,* and various brassicaceous species, including *B. oleracea*, *B. napus* and the Se hyperaccumulator *C. hupingshanensis* possess Φ thickenings near the endodermis [16,17,18,19,20]. *Pelargonium hortorum* has larger Φ thickenings at the hypodermis, in contrast to what has been observed in other plants [16,46]. We speculate that lignified Φ thickenings in the *M. glyptostroboides* roots might relate to the absorption of ions in aquatic habitats [17,18,20]. Mycorrhizae were present in the cortex and stele of *M. glyptostroboides* and might also participate in ion absorption [47,48,49]. Redivided or dilated cells were observed in the cortex and occasionally in the secondary phloem of *M. glyptostroboides* roots from aquatic habitats. This was similar to the dilated cortices in *Artemisia*, *Gentiana*, and *Tilia* tissues, as well as the dilated parenchyma in the aged roots of alligator weed [34,35,40,41]. Thus, dilated parenchyma may allow wetland plants to better tolerate being submerged.

In plants, air spaces retain oxygen under hypoxic and anoxic conditions to increase the likelihood of survival [22,23,24,25,28,50]. The roots of *M. glyptostroboides* from aquatic habitats had large, irregular air spaces in the cortex and phloem, in contrast to the roots of terrestrial *M. glyptostroboides*, which had narrower, smaller air spaces in the cortex and secondary phloem. However, these spaces were not observed by Basinger [4] in the well-preserved specimens of *M. milleri* roots or in the specimens of the studied *M. glyptostroboides* [9]. Air spaces have not been reported in the roots of other gymnosperms [10,11,12,13,14,15]. In other plants, including maize, wheat, and *H. sibthorpioides* [37,38,39,45], *Z. nicaraguensis*, and rice [25,26,38], the root aerenchyma is induced or increased during hydroponic growth. The schizogenous aerenchyma in the root cortex of aquatic alligator weed is larger than that in the root cortex of terrestrial alligator weed [35], similar to the differences in aerenchyma size observed here between aquatic and terrestrial *M. glyptostroboides*.

We found that an aquatic habitat resulted in multiple lignified Φ thickenings, larger air spaces, and dense suberin and lignin in the exodermis in *M. glyptostroboides*. Indeed, air spaces in the cortex and phloem of *M. glyptostroboides* roots provide oxygen for organs under anoxic conditions [25,26,28,29,31,32,33,34,35,50]. This was consistent with results in other species, including alligator weed, rice, maize, wheat, and *Z. nicaraguensis* [25,26,35,36,37,38,39]. The exodermis was not described by Basinger [4] in either fossil *M. milleri* or extant *M. glyptostroboides*, which is unsurprising due to histochemical limitations in this instance. The apoplastic barriers in the *M. glyptostroboides* fine adventitious roots consisted of the endodermis and exodermis with Casparian bands, suberin lamellae, lignin, and suberized cork. These barriers were similar to those observed in other wetland plants, such as alligator weed, *C. hupingshanensis*, *Typha*, *Iris*, *Phalaris*, and *Artemisia* [20,27,33,34,35,51,52,53].

In conclusion, the constitutive and induced apoplastic barriers that we observed in the *M. glyptostroboides* adventitious roots, as well as the lignified Φ thickenings and air spaces, might explain the ability of this species to thrive in both aquatic and terrestrial environments. The induced lignified Φ thickenings, which may improve ion absorption, may represent an adaptation to oligotrophic aquatic environments [17,18,20]. Future studies should quantify differences in additional parameters not fully explored here, such as the radicle structure, histochemistry, barrier permeability, and air space porosity between terrestrial and aquatic environments. Such results would help to predict the range of aquatic environments suitable for *M. glyptostroboides*. We predict that the phenotypic plasticity of the identified traits (Table 1) facilitates the growth of *M. glyptostroboides* in aquatic and terrestrial environments. This work may begin to explain how the rare plant *M. glyptostroboides* has survived despite abundant stressors, and may help to contextualize the taxonomy, evolution, and phylogeny of *M. glyptostroboides* within gymnosperms.

## 4. Materials and Methods

### 4.1. Sample Collection and Processing

We sampled the fine adventitious roots of terrestrial and aquatic *M. glyptostroboides*, then anatomically and histochemically analyzed the given specimens. The fine adventitious roots were collected during the summer, from plants located at the Qingtaiguan Forestry Station, Luotian County, Hubei Province, China. The climate at the Forestry Station is subtropical. The *M. glyptostroboides* forest at the forestry station is characterized by yellow-brown soil in terrestrial environments. Trees characterized as growing in aquatic environments grow along a river in sandy soil. These trees are seed-propagated and have been cultivated about 30 years. Terrestrial root samples were taken from roots growing in earth. Aquatic root samples were taken from constantly submerged roots stretching into the river. Root samples were taken about 5–14 m from the stem. More than 30 root samples were collected from three aquatic trees, and more than 30 root samples were collected from six terrestrial trees, including the terrestrial portions of the roots of three aquatic trees. Approximately 10–15 root samples were collected from each tree. Freshly-sampled adventitious roots were fixed in formaldehyde-alcohol-acetic acid (FAA) immediately following collection [54]. After fixation, root tissues were sectioned freehand, using a two-sided blade razor, under a stereoscope (JNOEC JSZ6, China). Sections were cut 5 mm, 10 mm, 15 mm, 20 mm, 25 mm, 30 mm, 50 mm, 100 mm, and 110 mm from the root tip to the base where the cortex had sloughed off. For each tree, 3–6 sections (from different samples) at each particular distance from the root tip were tested with each stain.

### 4.2. Histochemistry and Microscopy

Sections were stained with Sudan red 7B (SR7B) to test for suberin in the cell walls [55], and with phloroglucinol-HCl (Pg) to test for lignin in the cell walls [46,56]. We used berberine hemisulfate-aniline blue (BAB) and sulfuric acid digestion to test for Casparian bands and lignin in the cell walls [51,56,57], and used toluidine blue O (TBO) to visualize tissue structures [46,58]. All specimens were examined using bright-field microscopy with a Leica DME microscope, and photographed with a digital camera (Nikon E5400, Japan). Specimens stained with BAB were viewed under an Olympus IX71 epifluorescence microscope and photographed with a digital camera (RZ200C-21, China).

## Figures and Tables

**Table 1:** The structures and histochemistry of *Metasequoia glyptostroboides* grown in different habitats. Distances are from the root tips.

Organ Samples	Aquatic Habitat	Terrestrial Habitat
Primary structure	Endodermis and exodermis with Casparian bands, suberin, and lignin at 5–10 mm Dilated cortex, cortex sloughed off at 100 m Multiple lignified Φ thickenings in cortex Large air spaces in cortex and primary phloem Triarch to hexarch protoxylem and protophloem	Endodermis and exodermis with Casparian bands, suberin, and some lignin at 5 mm Cortex sloughed off at 25 mm Few lignified Φ thickenings in cortex narrow air spaces in cortex Diarch to triarch protoxylem and protophloem
Secondary structure	Secondary structure begins at 10–15 mm Secondary phloem and phellogen flared in places Exodermis has dense suberin and lignin Large air spaces in cortex and cork dilated parenchyma	Secondary structure begins at 10–15 mm No evidence of flaring in secondary phloem or phellogen Exodermis has typical suberin and lignin Narrow air spaces in cortex Undilated parenchyma

## Supplementary Materials

The following are available online at https://www.mdpi.com/2223-7747/8/11/501/s1, Figure S1: Phellogen close to the pericycle cork and under lysigenous primary phloem, Figure S2: Phellogen under lysigenous primary phloem.
