# Supplementary material for: Phenotypic Plasticity in the Structure of Fine Adventitious Metasequoia glyptostroboides Roots Allows Adaptation to Aquatic and Terrestrial Environments
Source: Plants (Basel). 2019 Nov 14;8(11):501. doi: 10.3390/plants8110501 (PMC6918158; doi:10.3390/plants8110501)
Supplement: Supplementary file 1 [file plants-08-00501-s001.pdf]

plants

Supplementary:

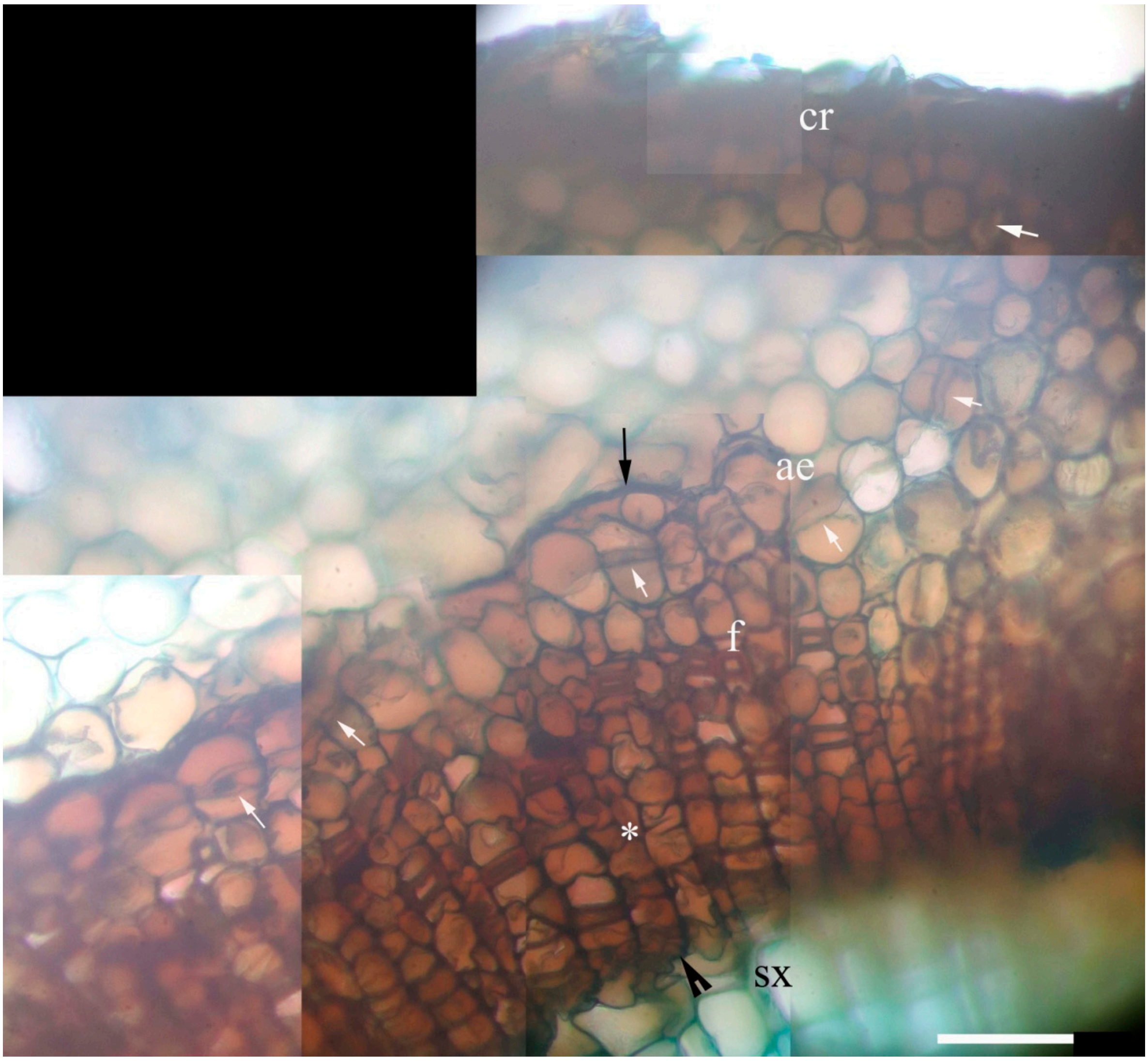

Figure S1. Phellogen close to the pericycle cork and under lysigenous primary phloem. Match to Fig. 2C, D, secondary xylem, vascular cambium (below arrowhead), phellogen (arrows), lysigenous primary phloem (black arrow), phloem aerenchyma, dilated parenchyma (*), phloem fibers, pericycle cork, staining: TBO; scale bars = 50 µm.

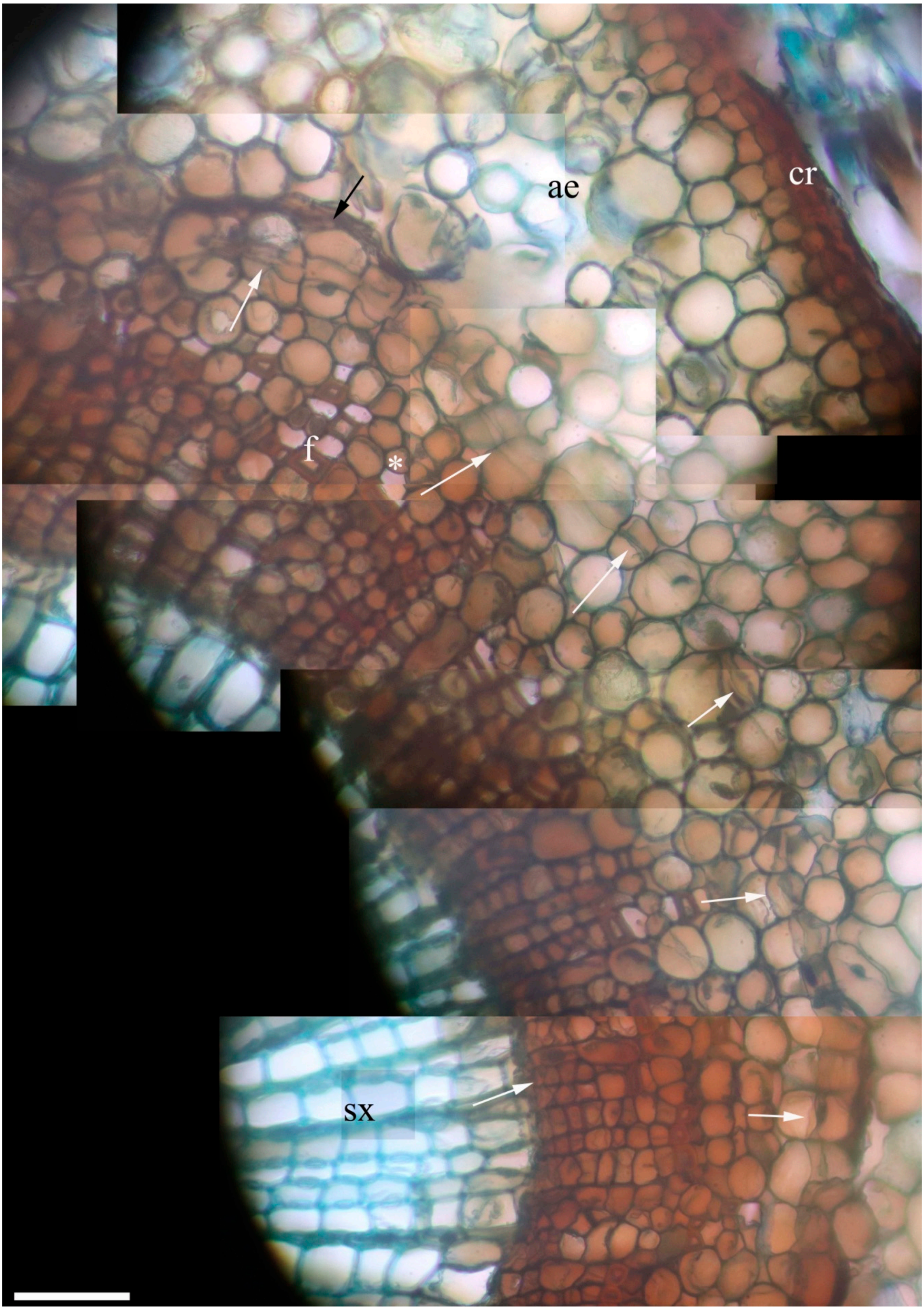

Figure S2. Phellogen under lysigenous primary phloem. Match to Fig. 2C, D, secondary xylem, vascular cambium (below arrow), phellogen (arrows), lysigenous primary phloem (black arrow), phloem aerenchyma, dilated parenchyma (*), phloem fibers, pericycle cork, staining: TBO; scale bars = 50 μm.
